# Glycoprotein nonmetastatic melanoma protein B accelerates tumorigenesis of cervical cancer *in vitro* by regulating the Wnt/β-catenin pathway

**DOI:** 10.1590/1414-431X20187567

**Published:** 2018-11-23

**Authors:** Shuxiang Xu, Yingying Fan, Dongping Li, Yan Liu, Xu Chen

**Affiliations:** Department of Obstetrics and Gynecology, Huashan Hospital North, Fudan University, Shanghai, China

**Keywords:** Cervical cancer, GPNMB, Tumorigenesis, MMPs, Wnt/β-catenin

## Abstract

Cervical cancer is one of the most common cancers among women around the world. However, the underlying mechanism involved in cervical cancer progression is incompletely known. In the present study, we determined the role of glycoprotein nonmetastatic melanoma protein B (GPNMB) in tumorigenesis of cervical cancer. According to the GEO database, we found that GPNMB expression was significantly higher in cervical cancer than in normal cervix epithelium. A similar pattern was observed in GPNMB expression in cultured cervical cancer cells and normal cervical epithelial cells. Compared with the control, GPNMB knockdown significantly decreased the proliferation and migration capacity, but enhanced the apoptosis capacity of SiHa and HeLa cells. Additionally, the activity of MMP-2 and MMP-9 were aberrantly increased in SiHa and HeLa cells compared with normal cervical epithelial cells, whereas their activities were strongly inhibited by GPNMB siRNA. Furthermore, Wnt/β-catenin signaling was activated by GPNMB in SiHa and HeLa cells. Increased MMP-2/MMP-9 expression was suppressed by Dkk-1, inhibitor of Wnt/β-catenin signaling, while it was enhanced by stimulator BIO. The proliferation, migration, and apoptosis capacity of HeLa cells were found to be affected by Dkk-1 and BIO to different extents. In conclusion, we demonstrated that GPNMB contributed to the tumorigenesis of cervical cancer, at least in part, by regulating MMP-2/MMP-9 activity in tumor cells via activation of canonical Wnt/β-catenin signaling. This might be a potential therapeutic target for treating human cervical cancer.

## Introduction

Cervical cancer is one of the most common malignant cancers among women around the world. Cervical intraepithelial neoplasia, transformed from normal cervical epithelium, is the major pathological characteristic, followed by the subsequent transformation to invasive cervical cancer (1). There are three principal histologic types of cervical cancer: adenocarcinoma, squamous carcinoma, and adeno-squamous carcinoma. Squamous carcinoma is the most common in patients with cervical cancer, including grade I (highly differentiated), grade II (moderately differentiated), and grade III (lowly differentiated). Infection by human papillomaviruses has been identified as the single most high-risk factor associated with human cervical cancer (2,3). However, the papillomaviruses infection alone is unlikely to cause invasive cervical carcinoma. Increasing evidence suggests that there are many unidentified genetic alterations involved in the tumorigenesis of cervical cancer (4). Hence, further understanding is necessary to improve therapeutic strategies in human cervical cancer.

Glycoprotein nonmetastatic melanoma protein B (GPNMB), a type I transmembrane glycoprotein, is comprised of three domains: single transmembrane domain, extracellular domain (464 amino acids), and short cytoplasmic domain (53 amino acids) (5). GPNMB is expressed in various normal tissues, including skin, bone, and hematopoietic system, and is involved in multiple biological processes, such as cell differentiation, proliferation, and inflammation (6). The dysregulation of GPNMB expression has been reported in many types of cancer, including hepatocellular carcinoma, malignant gliomas, and metastatic prostate cancer, and it is associated with the invasion and metastasis of tumor cells (7,8). In gynecological cancers, GPNMB also plays an important role in the progression of breast tumors and the metastasis of breast cancer (9,10). Additionally, GPNMB was recently identified as the specific marker and potential molecular therapeutic target in some cancers (8,11). However, the underlying role and mechanism of GPNMB in tumorigenesis of human cervical cancer remain incompletely known.

In the present study, we aimed to investigate the role of GPNMB in cervical cancer by determining the expression of GPNMB in cervical cancer and cells. Furthermore, we assessed *in vitro* its action in regulating matrix metalloproteinases (MMP)-2/MMP-9 activity via the Wnt/β-catenin pathway.

## Material and Methods

### Cell culture

Cervical cancer cells (HeLa, ME-180, and SiHa cells) were purchased from ATCC (Manassas, USA), and cultured in RPMI 1640 medium containing 10% fetal bovine serum (FBS, Invitrogen, USA) and 1% penicillin/streptomycin (Gibco/Invitrogen) at 37°C in a 5% CO_2_ atmosphere in an incubator. Normal cervical epithelial cells were obtained from ATCC, and maintained in DMEM medium supplemented with 10% FBS and 1% penicillin/streptomycin at 37°C.

### Cell transfection

The siRNA targeting GPNMB was obtained from Takara (China). Cells were grown to approximately 80% confluence, and transfected with GPNMB siRNA or negative control siRNA by Lipofectamine 3000 (Invitrogen) in accordance with the manufacturer's instructions. After 48 h, the transfection efficacy was evaluated by RT-PCR.

### Quantitative RT-PCR

Trizol (Sigma, USA) was used to obtain the total RNA from cells. The mRNA expression of GPNMB was detected using a RT-PCR kit (Takara) according to the manufacturer's instructions. The primers used were: GPNMB: 5′-AGAGTCAAGCCCTGACTGGC-3′ (forward) and 5′-GAAGAGTGGGTTCCCAGTCA-3′ (reverse). Ct values of GAPDH were used as the internal control, and relative expression of target genes are reported using 2^-△△CT^ method.

### Western blotting

Total proteins were obtained from cell lysates using lysis buffer (Bioss, China), and nuclear proteins were collected by Nuclear and Cytoplasmic Extraction Kit (Thermo Scientific, USA). Equal amounts of proteins were separated by SDS-PAGE, and electrophoretically transferred to a PVDF membrane. The membranes were incubated with primary antibodies (anti-GPNMB 1:2000 dilution, R&D Systems, USA; anti-MMP-2, MMP-9 1:1000 dilution, Abcam; anti-β-catenin 1:1000 dilution, Santa Cruz, USA; mouse anti-β-actin and LaminB1, 1:2000 dilution, Abcam, UK) at 4°C overnight, followed by treatment with secondary antibodies for 1 h at room temperature. Bands were then visualized by ECL (Amersham Pharmacia GE, China), and analyzed using Image Quant software (Amersham BioSciences, USA).

### Cell proliferation assay

Cell proliferation assay was performed using CCK-8/WST-8 Kit (Bioroot, China). Briefly, transfected cells (1×10^5^ cells/mL) were cultured in 96-well plates for 12, 24, 48, and 72 h, respectively. Then, 10 μL of CCK-8 solution was added to each well, and were incubated for another 4 h at 37°C. Absorbance of cells at 450 nm was tested using an ELISA plate reader (Amersham Pharmacia GE).

### Cell apoptosis assay

Cell apoptosis assay was performed by flow cytometric analysis using an annexin V-fluorescein isothiocyanate kit (BD Biosciences, Germany). In brief, cells were kept in serum-free medium for 16 h, followed by harvesting with ice-cold phosphate-buffered solution and re-suspending with binding buffer. Then, cells (5×10^6^ cells/well) were stained with annexin V-fluorescein isothiocyanate (0.5 μg/mL) and propidium iodide (0.6 μg/mL) for 15 min in the dark at room temperature, and immediately analyzed on a FACS Calibur^TM^ system (Becton Dickinson, USA).

### Cell migration assay

DMEM (600 μL) medium containing 10% FBS was added into the lower compartment of the chambers. Cells (5×105 cells/mL) were mixed with growth factor-free DMEM and kept in the upper chamber for 4 h. Then, cells were fixed with 90% ethanol and stained with 0.05% crystal violet for 15 min. Non-transmigrated cells were gently scraped off using a cotton swab and migrated cells on the lower surface of the membrane were counted in five fields per chamber.

### Gelatin zymography

Gelatin zymography assay was performed to evaluate the activity of MMP-2 and MMP-9 as previously described (12). In brief, equal amounts of proteins collected from the conditioned media of cultured cells were separated by SDS-PAGE containing 0.1% gelatin at 4°C. Then, gels were washed with 2.5% Triton X-100 for 30 min, and treated with substrate buffer at 37°C. After staining and destaining, bands was analyzed by Image Quant software.

### Statistical analysis

Cervical cancer-related raw data were downloaded from GEO database. A comprehensive analysis of GPNMB gene expression was performed based on the GDS3233 profiles containing 28 cervical cancer cases and 21 normal cervix epithelium cases. Data are reported as means±SE. Statistical analysis was performed by GraphPad Prism 6 (USA). The differences between groups were assessed by one-way ANOVA. P<0.05 was considered to be statistically significant.

## Results

### GPNMB was aberrantly expressed in human cervical cancer tissues and cells

**Figure 1.**
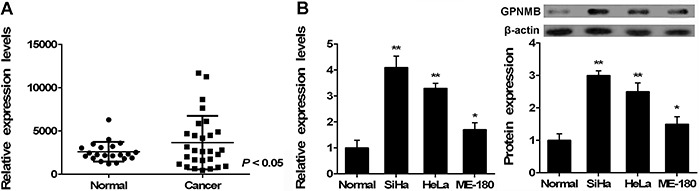
*A*, Expression of glycoprotein nonmetastatic melanoma protein B (GPNMB) in human cervical cancer patients as well as in normal cervix epithelium from GEO database. *B*, RT-PCR and western blot assay were performed to evaluate the mRNA and protein expressions of GPNMB in human cervical cancer cells SiHa, HeLa, and ME-180 cells, as well as in normal cervical epithelial cells. Data are reported as means±SE. *P<0.05, **P<0.01 *vs* Normal (ANOVA).

Based on the GEO database, GPNMB expression was significantly up-regulated in cervical cancer compared with normal cervix epithelium (Figure 1A). We further measured the expression levels of GPNMB in human cervical cancer HeLa, ME-180, and SiHa cells. Results indicated that GPNMB expression was kept at a low level in normal cervical epithelial cells, but was aberrantly increased in all cervical cancer cells (Figure 1B). These results suggested that dysregulation of GPNMB expression might be associated with the tumorigenesis of human cervical cancer.

### GPNMB accelerated the tumorigenesis of cervical cancer *in vitro*

**Figure 2.**
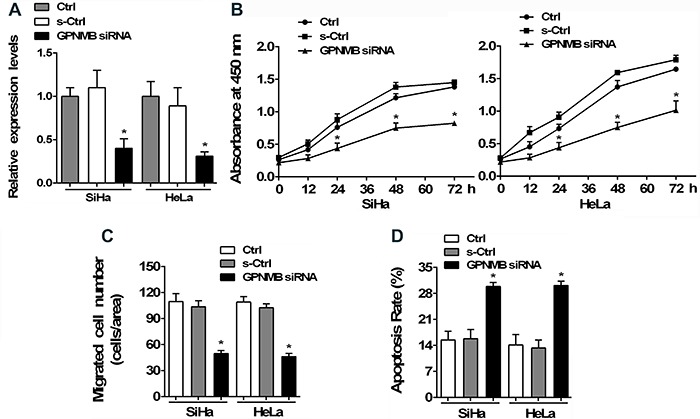
SiHa and HeLa cells were cultured in normal medium (Ctrl), then transfected with glycoprotein nonmetastatic melanoma protein B (GPNMB) siRNA or negative control siRNA (s-Ctrl). *A*, RT-PCR assay was performed to evaluate the transfection efficacy of specific GPNMB siRNA in cervical cancer cells. *B*, CCK-8 assay was conducted to clarify the proliferation of SiHa and HeLa cells after treatment with siRNA or not. *C*, Migration capacity of cells evaluated by Transwell Boyden chambers. *D*, Apoptosis of cells was measured by flow cytometry. Data are reported as means±SE. *P<0.05 *vs* Ctrl (ANOVA).

To further clarify the underlying role of GPNMB dysfunction in tumorigenesis of cervical cancer, we silenced GPNMB gene by specific siRNA in SiHa and HeLa cells. Transfection efficacy of the specific GPNMB siRNA was evaluated by RT-PCR assay (Figure 2A). Compared to the control, the proliferation capacity of SiHa and HeLa cells was significantly decreased by GPNMB inhibition (Figure 2B). Similarly, knockdown of endogenous GPNMB reduced the migration capacity of SiHa and HeLa cells (Figure 2C). On the contrary, flow cytometric analysis showed that the apoptosis capacity of SiHa and HeLa cells was enhanced by GPNMB siRNA compared to the control (Figure 2D). Taken together, our results indicated that dysregulation of GPNMB expression played a positive role in the motility of cancer cells, contributing to the tumorigenesis of cervical cancer.

### GPNMB regulated the expression and activity of MMP-2 and MMP-9 in cervical cancer cells

**Figure 3.**
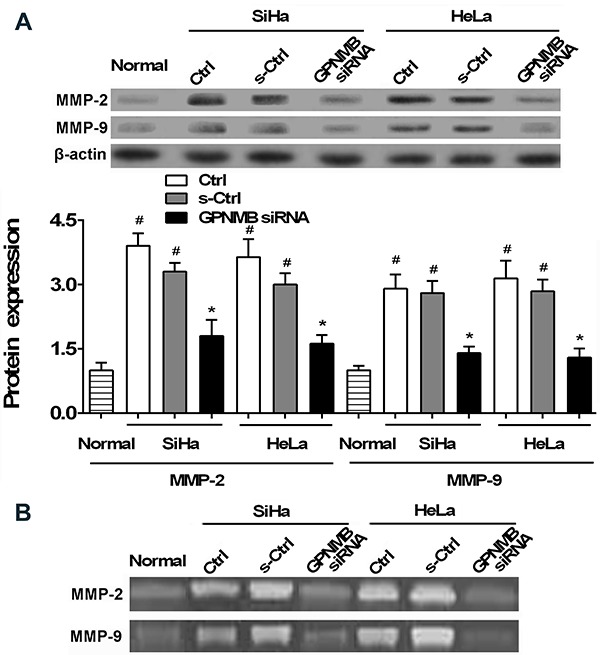
SiHa and HeLa cells were cultured in normal medium (Ctrl), then transfected with glycoprotein nonmetastatic melanoma protein B (GPNMB) siRNA or negative control siRNA (s-Ctrl). *A*, Expression of MMP-2 and MMP-9 were measured by western blotting in SiHa and HeLa cells as well as in normal cervical epithelial cells (Normal). *B*, Gelatin zymography assay was performed to evaluate the activity of MMP-2 and MMP-9 in conditioned media of cells. Data are reported as means±SE. ^#^P<0.05 *vs* Normal; *P<0.05 *vs* Ctrl (ANOVA).

It is known that MMPs are crucial mediators and participators in tumorigenesis and metastasis of cancer (13–15). Compared with normal cervical epithelial cells, MMP-2 and MMP-9 expression was aberrantly increased in SiHa and HeLa cells, and significantly decreased after GPNMB siRNA treatment (Figure 3A).

Results from the gelatin zymography assay showed that GPNMB knockdown greatly down-regulated the levels of MMP-2 and MMP-9 in conditioned media of cultured SiHa and HeLa cells compared with the control (Figure 3B). These data demonstrated that GPNMB played a positive role in cervical cancer progression, at least in part, by regulating the expression and activity of MMP-2/MMP-9 in tumor cells.

### Wnt/**β**-catenin pathway is involved in the GPNMB-mediated cervical cancer tumorigenesis *in vitro*

**Figure 4.**
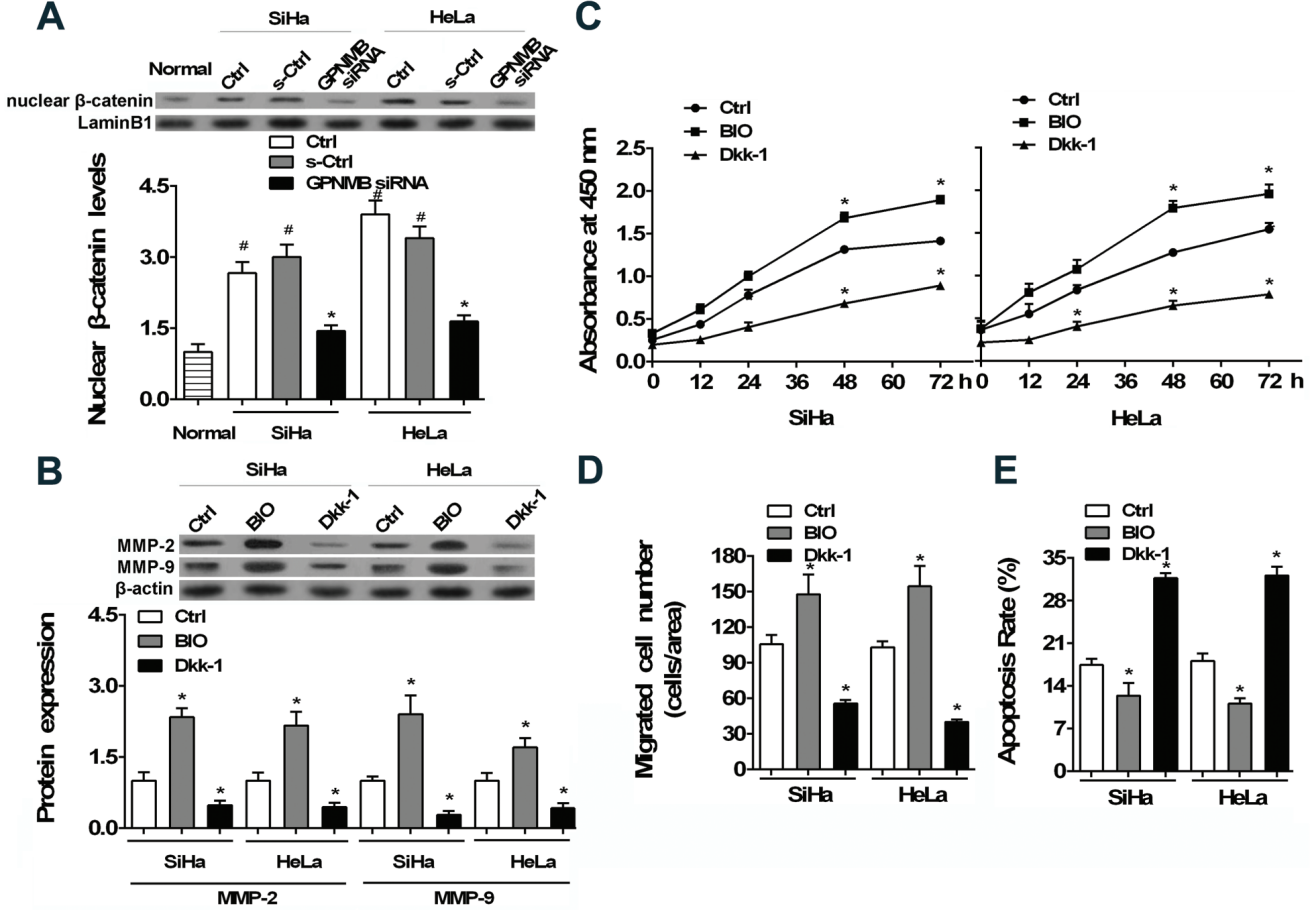
Association between Wnt/β-catenin pathway and glycoprotein nonmetastatic melanoma protein B (GPNMB)-mediated cervical cancer tumorigenesis. SiHa and HeLa cells were cultured in normal medium (Ctrl), then transfected with GPNMB siRNA or negative control siRNA (s-Ctrl). *A*, Expression of nuclear β-catenin measured by western blotting in SiHa and HeLa cells as well as in normal cervical epithelial cells. *B*, Cells were treated by 100 ng/ml Dkk-1 or 1 nM BIO for 48 h or not, and matrix metalloproteinases (MMP)-2 and MMP-9 expressions were then measured by western blotting. *C*, CCK-8 assay was conducted to clarify the proliferation of SiHa and HeLa cells after treatment with Dkk-1 or BIO. *D*, Migration capacity of cells tested by Transwell Boyden chambers. *E*, Apoptosis of cells measured by flow cytometric analysis. Data are reported as means±SE. ^#^P<0.05 *vs* Normal; *P<0.05 *vs* Ctrl (ANOVA).

Wnt/β-catenin pathway has been identified as an important regulatory approach in the progression of many diseases, including cervical cancer and other cancers (16,17). We investigated whether Wnt/β-catenin pathway was associated with GPNMB-mediated cervical cancer tumorigenesis *in vitro*. Results showed that nuclear β-catenin, the indicator of Wnt/β-catenin signaling, was kept at low level in normal cervical epithelial cells, but was significantly increased in cultured SiHa and HeLa cells, as shown in Figure 4A. Conversely, the increased nuclear β-catenin was attenuated by GPNMB suppression.

In a subsequent experiment, we used BIO, a stimulator of Wnt/β-catenin pathway, and Dkk-1, an inhibitor of Wnt/β-catenin signaling, to further determine the underlying role of Wnt/β-catenin. It was found that increased MMP-2/MMP-9 expression was suppressed by inhibitor Dkk-1, while it was enhanced by the stimulator BIO (Figure 4B). Besides, the proliferation, migration, and apoptosis capacity of HeLa cells were also affected by Dkk-1 and BIO to different extents (Figure 4C to E). These results demonstrated that GPNMB dysregulation contributed to the tumorigenesis of cervical cancer, at least in part, by regulating the activity of MMP-2/MMP-9 in tumor cells via activation of the Wnt/β-catenin pathway.

## Discussion

GPNMB is a type I transmembrane glycoprotein that is expressed in various tissues and is involved in multiple physiological processes (6). Dysregulation of GPNMB expression has been identified to be associated with pathological progression, including aggressive cancers (8,9). GPNMB is highly expressed in high-grade glioma, and its specific scFv antibodies and immunotoxins present feasibility as reagents in the immunotherapy of malignant gliomas and melanomas (11). In breast cancer, a common gynecological malignancy, overexpressed GPNMB plays roles as mediator in metastasis to bone as well as in aggressively lung-metastatic breast cancer (9,10). Dysfunction of GPNMB has been recognized in metastatic prostate cancer, hepatocellular carcinoma, and other cancers (7,8). Additionally, GPNMB was recently identified as the specific marker and potential molecular therapeutic target in some cancers (8,11). In the present study, we found that GPNMB expression was significantly increased in cervical cancer. Notably, we determined the expression profile of GPNMB in normal cervical epithelial cells, which were cultured in medium containing FBS and controlled time to avoid possible effects of differentiation. Results indicated that GPNMB expression was kept at low levels in normal cervical epithelial cells, implying the probable involvement of GPNMB in the progression of cervical cancer.

The pathology involved in cervical cancer progression is associated with cervical intraepithelial neoplasia of normal cervical epithelium. Proliferation, invasion, and metastasis of tumor cells represent the major cellular events in tumorigenesis of invasive cervical cancer (2,4). Previous studies demonstrated that dysfunction of GPNMB has an important regulatory role in alteration of cellular function in tumor cells. Zhang et al. (18) reported that silencing GPNMB by siRNA could attenuate the formation of melanosomes in melanocytes in a microphthalmia-associated transcription factor-independent manner. Fiorentini et al. found that GPNMB could enhance the invasiveness of human metastatic prostate cancer cells, and siRNA-induced GPNMB silencing inhibits the proliferation and migration of cancer cells *in vitro* (19). By employing GPNMB-specific scFv antibodies or immunotoxins, there was significant anti-tumor activity observed in GPNMB-expressing glioma and malignant melanoma cells *in vitro* as well as malignant glioma xenografts in mouse and melanoma neoplastic meningitis *in vivo* model (11). In a recent report, Zhang YX et al. (20) determined that GPNMB plays a crucial role in the progression of bladder cancer and GPNMB knockout can inhibit the proliferation, migration, and invasion of bladder cancer cells. Herein, we also determined that GPNMB can accelerate the motility of cancer cells *in vitro*, which might contribute to tumorigenesis of cervical cancer.

The MMPs family is a crucial mediator of tumorigenesis and plays key roles in tumor invasion and metastasis by degrading the extracellular matrix components and destroying the histologic barrier of tumor metastasis. Among them, MMP-2 and MMP-9 are the major catabolic enzymes of type IV collagen to degrade the basic component of basement membranes. Prior studies indicate that highly expressed MMP-2 can facilitate the activation of gelatinase MMP-9, contributing to the invasion and metastasis of cancer cells (21).

In cervical cancer, dysregulation of MMP-2 and MMP-9 has been widely reported. MMP-2 and MMP-9 expression and activation can be affected by various factors, including cytokines, mitogens, inducers, inhibitors, E6/E7 oncoproteins of human papillomavirus 16 (HPV16), and others (22,23). It was reported that the activation of MMP-2 in cervical cancer tissue could be mediated by a functional complex consisting of α(v)β3 integrin/membrane type-1 metalloproteinase-2 (MT1-MMP)/tissue inhibitor of metalloproteinase-2 (TIMP-2) on tumor cell surface (24). Additionally, MMP-2 and MMP-9 expression could also be regulated by histone deacetylases (HDACs), a key enzyme of epigenetic regulation, to affect cervical cancer metastasis (25). We herein determined that expression and activity of MMP-2 and MMP-9 were aberrantly increased, but were significantly decreased by GPNMB siRNA in cervical cancer cells *in vitro*. These findings are in line with previous studies demonstrating that GPNMB increases the invasion capacity of metastatic prostate cancer cells by activating MMP-2 and MMP-9 (19). The high expression and activity of MMP-2 and MMP-9 have been recognized to be correlated with HPV presence in cervical cancer and the present results were obtained in HPV positive cervical cancer cells. It would be very interesting to clarify whether there are possible associations among GPNMB, MMP-2/MMP-9, and HPV oncogenes, and whether GPNMB plays a role in HPV-negative cervical cancer cells in future study (26–28).

Furthermore, Wnt/β-catenin pathway has been implicated in tumorigenesis (29–31). In cervical cancer, Lin et al. (32) reported that aberrant Wnt/β-catenin activation is involved in the modulation of secreted frizzled-related proteins (SFRPs) to cell growth and invasion. Through canonical Wnt/β-catenin pathway, sulfiredoxin might be an oncoprotein to promote the metastasis of cervical cancer (33). Activation of Wnt/β-catenin signaling might a play positive role in the pathogenesis of cervical cancer by up-regulating Twist (34). Additionally, Wnt/β-catenin pathway is also correlated with the expression and activity of MMP-2 and MMP-9, co-contributing to cancer progression (35,36). In this study, we found that Wnt/β-catenin pathway was activated, but was attenuated by GPNMB siRNA in cervical cancer cells and the activated Wnt/β-catenin signaling was associated with GPNMB-mediated cervical cancer tumorigenesis *in vitro*. These findings are consistent with a previous report in which GPNMB was able to enhance the motility and angiogenesis in glioma by regulating the Wnt/β-catenin pathway, implying the important role of GPNMB in the progression of multiple cancers (37).

In conclusion, we demonstrated that GPNMB expression was aberrantly regulated in cervical cancer. Furthermore, we found that GPNMB accelerated the tumorigenesis of cervical cancer *in vitro* by regulating MMP-2/MMP-9 activity via the Wnt/β-catenin pathway. These findings suggested a novel role of GPNMB in the progression of cervical cancer, and might be a possible target for treating cervical cancer.
